# The potential longevity-promoting hypoxic-hypercapnic environment as a measure for radioprotection

**DOI:** 10.1007/s10522-024-10129-3

**Published:** 2024-08-20

**Authors:** Elroei David, Marina Wolfson, Khachik K. Muradian, Vadim E. Fraifeld

**Affiliations:** 1 Nuclear Research Center Negev (NRCN), P.O. Box 9001, 8419001 Beer-Sheva, Israel; 2 The Shraga Segal Department of Microbiology, Immunology and Genetics, Faculty of Health Sciences, Center for Multidisciplinary Research on Aging, Ben-Gurion University of the Negev, 8410501 Beer Sheva, Israel; 3 Department of Aging Biology and Experimental Life Extension, Institute of Gerontology, NAMS of Ukraine, Kiev, 04114 Ukraine

**Keywords:** Ionizing radiation, Hypoxia, Hypercapnia, Aging, Radioprotection, Hormesis

## Abstract

Many biological mechanisms of aging well converge with radiation’s biological effects. We used scientific insights from the field of aging to establish a novel *hypoxic-hypercapnic environment (HHE) concept for radioprotection*. According to this concept, HHE which possesses an anti-aging and longevity-promoting potential, should also act as a radiomitigator and radioprotector. As such, it might contribute greatly to the safety and wellbeing of individuals exposed to high levels of radiation, whether in planned events (e.g. astronauts) or in unplanned events (e.g. first responders in nuclear accidents).

*We cannot solve our problems with the same thinking we used when we created them*

—Albert Einstein

## Introduction

Aging and rejuvenation research gave a push to the development of various promising applications which, on the first view, seem to be quite far from biogerontology. Indeed, several modern biomedical fields have originated or greatly influenced from rejuvenation and lifespan extension studies. Well-known examples include hormone replacement therapy, probiotic diets, tissue transplantations in humans, tissue engineering, cell therapy, etc. (Stambler 2014). Here, we introduce a novel hypothesis which utilizes insights driven from the field of aging for the benefits of radioprotection research.

### Ionizing radiation, radioprotectors and radiomitigators

Exposure to harmful ionizing radiation (IR) could be the result of planned or unplanned events. Unplanned events include nuclear or radiological accidents (e.g. Chernobyl, Fokushima), potential terrorist events, and incidents of lost, stolen or misplaced radioactive sources (National Academies of Sciences 2021; US Department of Homeland Security 2007). Planned events which involve exposure to IR, include mainly medical diagnostics, interventional radiology and nuclear medicine—for both the general public, but to a far more extent for medical professionals (e.g. radiologists, CT-technicians). Other planned events are also air travels, essentially for aircrew and frequent flyers, and eventually astronauts—who receive the highest doses of all.

The collective effective dose and effective dose per individual in the U.S., originating from medical sources, raised more than three folds from the early 1980s to 2006 (National Council on Radiation Protection and Measurements 2009). Medical professionals are at much higher risk, as they are continuously and routinely exposed to both primary and scattered radiation during various radiological procedures (Shafiee et al. 2020).

In air travels, while the exposure dose is relatively low, it depends on two major factors: the duration of the flight, and the altitude. Namely, the longer the flight and the higher the flight altitude are, the higher is the exposure (National Council on Radiation Protection and Measurements 2009). Thus, frequent flyers and aircrew are much more exposed to cumulative higher doses of IR than the general public. Seven to eight travels back and forth from Los Angeles to western Europe are enough to reach the International Commission on Radiological Protection (ICRP) dose limit (Alvarez et al. 2016).

In space, astronauts are exposed to protons and high energy and charge (HZE) ions, as well as to secondary radiation produced by nuclear reactions in spacecraft (Cucinotta et al. 2006; Furukawa et al. 2020). While spaceflight in low Earth orbit (e.g. missions on the International Space Station), are partially protected by the solid shielding of the planet and Earth’s magnetic field, missions to farther areas, and for longer times, (e.g. Apollo missions, proposed missions to the Moon or Mars) might lead to much higher exposure doses (Cucinotta et al. 2006; Furukawa et al. 2020). For instance, the ICRP’s guidelines dictate a maximum occupational effective dose which should not exceed 20 mSv/year, with no annual effective dosage exceeding 50 mSv/year (Akram et al. 2022). Yet, a possible mission to Mars could last as long as 3 years, resulting in a whole-body dose of 1000 mSv or even more (Furukawa et al. 2020; Wilson et al. 1995). In fact, the effective dose of the past space missions Skylab, Mir and ISS of the National Aeronautics and Space Administration (NASA), reached in some personnel to more than 100 mSv (Cucinotta et al. 2008).

The availability and development of appropriate medical countermeasures for radiation (MCM) is still a substantial unmet medical need, which has been categorized by the U.S. government as a high priority area (Singh et al. 2017). Most recently, the U.S. Food and Drug Administration (FDA) stated that improving the capabilities to address radiological and nuclear emergencies is a national priority, and that drugs developed for Acute Radiation Syndrome may be eligible for certain FDA expedited programs (e.g., fast track and priority review) or other FDA programs (e.g., orphan drug designation) (US Food and Drug Administration 2023).

Depending on the time of delivery, pre or post exposure, MCM could generally be divided into three categories: *radioprotectors*, which are given for prophylaxis ahead of exposure; *radiomitigators*, which are given during or soon after exposure, and *treatment* of radiation injuries (Stone et al. 2004; Obrador et al. 2022). It is sometimes difficult to distinguish between radioprotectors and radiomitigators, since both could activate the same biological mechanisms (e.g., antioxidation) (Obrador et al. 2022).

Despite years of planning and billions of dollars in training and equipment, it seems that the level of preparedness of first responders in case of nuclear or radiological emergencies is still insufficient (Ingram 2018). This is true particularly in MCM designed for protecting against and mitigating exposure-related consequent morbidity and/or mortality responses (Singh et al. 2015). Here, we introduce a novel approach to meet the challenge of radioprotection/radiomitigation, applying lessons learned from the field of aging.

### Radiation and aging

**Fig. 1:**
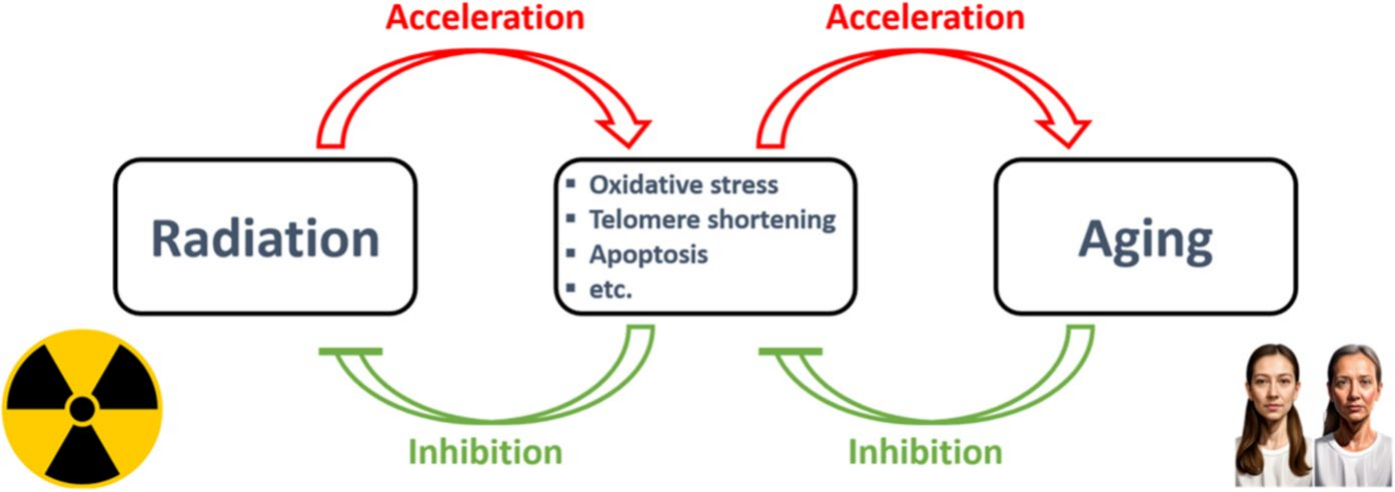
The logics of the current hypothesis. Since IR induces multiple damage mechanisms, which in turn accelerates aging, it is reasonable that inhibition of aging-related damage should attenuate IR harmful effects

Since the beginning of the atomic era, there has been an extensive investigation on the effects of IR on longevity. Exposure to IR has long been known as a model for accelerated aging, and multiple formulas has been introduced over the years to connect radiation levels with aging (Mewissen et al. 1957; Bertell 1977; Moskalev et al 2013; Wada et al. 2022). Based on recent surveys of updated experimental data, many biological mechanisms of aging evidently well converge with radiation’s biological effects (Richardson 2009; Tong & Hei 2020; Al-Jumayli et al. 2022). These include oxidation stress, chromosomal damage, telomere shortening, apoptosis, cellular senescence, inflammation, and stem cell exhaustion—all result in tissue damage. All in all, damage is recognized as a crucial factor in aging (Gladyshev et al. 2021), as well as in IR exposure. If so, it is only reasonable to hypothesize that if IR-induced damage leads to accelerated aging, inhibition of aging-related damage should attenuate IR harmful effects. Thus, anti-aging and longevity-promoting factors could also serve as radioprotectors and/or radiomitigators against radiation-induced damages (Fig. 1).

### Low metabolic rate and body temperature promote longevity

Two major factors which are well known to promote lifespan extension are low metabolic rate (MR) and low core body temperature (T_b_) (Houtkooper et al. 2010; Lehmann et al. 2013). In fact, these factors were recently suggested to be major players in the increasing human health and longevity over the past 157 years (Protsiv et al. 2020). While the relationship between MR and T_b_ and their interdependence with regard to longevity are still not fully clear (Carrillo & Flouris 2011), recent findings highlight the possible dominant role of T_b_ (Zhao et al. 2022). Keil et al. (2015) reviewed the literature concerning the influence of T_b_ on aging and longevity, and offer several possible mechanisms of temperature life-extending effects. For instance, Ames dwarf mice, which live much longer than normal size controls, maintain lower T_b_, but not lower metabolic rate, than their normal siblings (Hunter et al. 1999; Mattison et al. 2000). Likewise, Conti et al. (2006) found that transgenic mice with only 0.3–0.5 °C reduction of the core body temperature had a greater median life span in both males and females. The naked mole-rats (NMRs; *Heterocephalus glaber*), which are the longest-lived rodents with a maximum lifespan exceeding 37 years, have much lower MR and T_b_ compared to rodents with a similar body mass (Oka et al. 2023). Another interesting species is the Bowhead whale (*Balaena mysticetus*)—possibly the longest-living mammal, which is estimated to live over 200 years (Tacutu et al. 2018). It was found that bowhead whales have lower T_b_ compared to other whales with a shorter life expectancy (Lagunas-Rangel 2021). It is important to note that the beneficial effect of low T_b_ on lifespan extension was found in both ectothermic and endothermic organisms (Flouris et al. 2014). Roth et al. (2002) compared the survival of healthy men in the Baltimore Longitudinal Study of Aging (BLSA), and revealed that men with lower caloric restriction markers exhibited lower core body temperature and higher survival than their counterparts. In primates, caloric restriction essentially leads to lower T_b_ and also lowers MR (Roth et al. 2002). Various mechanisms were suggested to explain the possible role of T_b_ and MR in longevity (Keil et al. 2015; López-Otín 2016). Whatever the case, the results obtained indicate that low T_b_ and low MR, together or independently, can promote longevity. In view of the common mechanisms of aging and IR, it would be plausible to suggest that interventions leading to hypometabolism and hypothermia might also possess radioprotective activity. Do we have such means in hand?

### Hypoxic-hypercapnic environment lead to lower T_b_ and MR

Long-lasting and stable reduction of MR and T_b_ by non-genetic manipulations has been a technical challenge for a long time. Only recently, this issue was resolved by using self-induced hypoxic-hypercapnic environment (HHE) (Tolstun et al. 2020). This approach was based on the “pull and push back” concept of longevity and lifespan extension, introduced by Muradian (2013). According to this hypothesis, hypoxic state (decreased oxygen levels in body tissues) combined with hypercapnia (increased partial pressure of carbon dioxide), could create preconditions for reduction in MR and related T_b_. As a consequence, it could have the potential to reduce aging rate and promote longevity. One of the most famous examples is that of the naked mole-rat, which lives in nature in a high CO_2_/low O_2_ environment (Park et al. 2021; Amoroso et al. 2023). As mentioned above, NMRs are the longest-living rodents (Oka et al. 2023). Their core body temperature is approximately 32 °C compared to the ordinary 37 °C of mice, and their MR is about two-thirds of similarly sized rodents (Park et al. 2021; Amoroso et al. 2023). In addition, NMRs have pronounced resistance to several age-related diseases, including cancer, as well as to physical health deterioration in old age (Lin & Buffenstein 2021). Even in advanced age, NMRs maintain reproductive capabilities and other muscle and organ functions (Delaney et al. 2021). These remarkable effects could be attributed to a great extent to the HHE in which they live (Pamenter et al. 2022). This was further supported by Tolstun et al. (2020), which managed to induce metabolic remodeling in mice by creating artificial self-induced hypoxic-hypercapnic environment. The outcome was a significant and long-standing decrease in MR, T_b_, and food consumption—all are well recognized longevity-promoting factors (Tolstun et al. 2020). Indeed, HHE had recently been shown to increase lifespan in mice, and further suggested as a potential mean to extend life expectancy (Kulikov et al. 2019). HHE was shown to increase the tolerance to ischemia, and has a greater adaptogenic potential compared to hypoxia alone (reviewed by Tolstun et al. 2022). The benefits of scheduled hypometabolism and hypothermia induced by HHE in deep space manned missions was recently reviewed by Muradian et al. (2024). Considering the “Seven knowledge gaps in modern biogerontology” (Rattan 2024), the HHE hypothesis could, to some extent, fulfill the knowledge-gap related to homeodynamic space. Specifically, HHE could enhance health and survival ability in the elderly (Fraifeld and Muradian 2021). Maybe even more, the HHE hypothesis is relevant to the knowledge-gap of differentiating age-related changes, showing that hypoxia and hypercapnia are not only harmful, but may benefit health and prevent some aging-related pathologies. A summary of relevant papers on the beneficial health effects of HHE is provided in Table 1.

**Table 1:** A summary of relevant papers regarding the beneficial health effects of hypoxia-hypercapnia

Species	HHE effect	References
Mice	Repeated respiratory exercises with hypercapnic hypoxia increases lifespan in mice. In old mice hypercapnic hypoxia improved reproductive and cognitive functions, and increased motor and search activity, as well as physical stamina	Kulikov et al. (2019)
Rats	Mild hypercapnia has a protective effect against severe brain damage induced by cerebral hypoxia–ischemia	Vannucci et al. (1995, 2001)
Rats	Hypercapnia inhibits hypoxia-induced pulmonary vascular remodeling and right ventricular hypertrophy, reduces hypoxic pulmonary vasoconstriction, and protects against hypoxia-induced impairment of endothelial function	Ooi et al. (2000)
Rats	Hypercapnia protects erythrocytes against free radical damage induced by hypoxia, presumably by direct interaction of CO_2_ with free radical processes	Skoumalová et al. (2008)
Rats	HHE effectively prevented acute disturbances in cerebral circulation and stroke	Yakushev et al. (2008)
Rats	Mild and moderate hypercapnia is neuroprotective after cerebral ischemia	Zhou et al. (2010)
Rats	Combined exposure to hypercapnia and hypoxia provides maximum neuroprotective effect during focal ischemic brain injury	Tregub et al. (2015)
Rats	Hypercapnia attenuates the damage from ischemic brain injuries caused by mild or moderate hypoxia	Yang et al. 2016
Pigs	Hypercapnia related acidosis protects the brain of intra-uterine growth-restricted newborn piglets from hypoxia/reoxygenation induced injury	Barth et al. (1998)
Humans	Respiratory training with hypercapnic hypoxia showed improved neurological and neurophysiological outcomes in children with cerebral palsy. The authors conclude that hypercapnic hypoxia could be considered a method of improving the efficiency of standard therapy	Kulikov et al. (2022)

Following the logics that was presented above, it is reasonable to assume that potential longevity-promoting HHE might also have the potential to act as a radioprotector/radiomitigator, thus reducing the sensitivity to IR. One of the mechanisms of HHE radioprotective potential could be related to hormetic effects. Indeed, hypoxia and hypercapnia are potentially harmful factors. At certain levels, they could act as hormetins and induce hormetic effects (Mattson 2008; Yang et al. 2016; Bondy 2023), accompanied by cross-adaptive reactions (Rattan 2008; Calabrese and Agathokleous 2022), so that HHE might reduce harmful effects of IR.

### Evidence for putative radioprotection and radiomitigation effects of HHE

In the 1950-60 s, a series of papers in *Nature* highlighted the possible radioprotective effect of hypoxia (Thoday & Read 1947; Weiss 1959; Lindop & Rotblat 1960; Rothe et al. 1963; Larsson & Stenson 1965). Since the 1950s, it was well known that there is a negative correlation between oxygen and the sensitivity to radiation in biological systems (Gray 1957). Researches proved over and over that conditions of hypoxia result in a considerable and various radioprotective effects in plants (Thoday & Read 1947), mice and rats (Weiss 1959; Lindop & Rotblat 1960; Rothe et al. 1963; Larsson & Stenson 1965), and in other mammals (Dowdy et al. 1950; Jamieson & van den Brenk 1961). For instance, Lindop and Rotblat (1960) showed that the LD_50/30_ of mice exposed to conditions of hypoxia for less than one minute, increased by approximately 35%, from ~1100 Rads to 1500 Rads. In fact, Duncan and Nias (1977) reviewed this issue, and found that hypoxia has been found to reduce the pathological effects of radiation by a factor of 2–3 in various tissues. They concluded that no other chemical agent exhibited similar effectiveness, and that molecular oxygen is undoubtedly the most significant single factor which modifies radiosensitivity.

Another evidence comes from the phenomenon of *tumor hypoxia*. Tumor hypoxia is a common feature of the microenvironment in solid tumors, as cellular proliferation outgrows the blood supply. This leads to resistance to radiotherapy and results in a poorer clinical outcome (Graham and Unger 2018; Sørensen and Horsman 2020; Pietrobon and Marincola 2021; Brown 2002). Most interestingly, mouse embryonic and skin primary fibroblasts were found to be much more sensitive to radiation damage than those of the naked mole-rat (Zhao et al. 2018).

The importance of hypercapnia is that it has a protective effect against the free radical oxidative damage induced by both hypoxia (Ooi et al. 2000; Vannucci et al. 2001; Skoumalová et al. 2008; Table 1) and presumably also against the oxidative stress induced by IR (see Fig. 1).

## Concluding remarks

In view of the hypometabolic and hypothermic effects of HHE, it evidently has a high potential for being anti-aging and longevity-promoting intervention. Since aging shares common features with IR injury, HHE could presumably act as a radioprotector/radiomitigator. We believe that HHE might contribute greatly to the safety and wellbeing of individuals exposed to IR.

## Data Availability

No datasets were generated or analysed during the current study.

## Abbreviations

HHE: Hypoxic-hypercapnic environment
ICRP: International Commission on Radiological Protection
IR: Ionizing radiation
MCM: Medical countermeasures
MR: Metabolic rate
NMR: Naked mole-rat
T_b_: Body temperature
